# The impact of vocational rehabilitation on employment outcomes: A regression discontinuity approach

**DOI:** 10.5271/sjweh.4038

**Published:** 2022-08-31

**Authors:** Mikko Laaksonen, Ilari Ilmakunnas, Samuli Tuominen

**Affiliations:** 1 Finnish Centre for Pensions (ETK), 00065 Eläketurvakeskus, Helsinki, Finland

**Keywords:** income, occupational rehabilitation, quasi-experiment, unemployment

## Abstract

**Objectives:**

Since 2015, Finnish disability pension applicants who are rejected or receive a short-term temporary pension have, under certain conditions, also received a preliminary decision for vocational rehabilitation (VR). A key requirement for eligibility is a certain amount of earnings during the previous five years (€34 910.29 in 2017). We exploit this discontinuity to examine the impact of assignment to VR on labor market outcomes.

**Methods:**

All new disability pension applicants from 2015 to 2017 were included. Fuzzy regression discontinuity design was used to evaluate the impact of assignment to VR on employment, unemployment and earned income two years later among those close to the threshold (+/- €20 000) providing eligibility for the preliminary decision. Arguably, those just below and just above the earnings limit are similar to each other, allowing causal interpretation of the estimates.

**Results:**

For each of the employment outcomes, we found a modest effect in the expected direction at the income threshold, but there is considerable uncertainty in these findings. On average, exceeding the income limit increased the probability of employment by 7.6 percentage points, but the estimate was far from statistical significance. Unemployment became slightly less common and earned income slightly increased, but the estimates were also clearly statistically non-significant.

**Conclusions:**

We found no consistent evidence of the impact of assignment to VR on employment outcomes among low-income disability pension applicants. However, given the narrow and specific study population, this should not be taken as evidence of ineffectiveness of VR more generally.

In Western countries, a significant proportion of the working-age population suffers from chronic health problems, and many leave the labor market before their statutory retirement age (1, 2). It has been estimated that around 30% of workers aged 50–64 need urgent adjustments to their work to prevent early retirement and work disability (3). Being able to continue working despite health problems is important for enhancing one’s personal well-being, promoting economic self-sufficiency, and preventing old-age poverty. From a societal perspective, keeping people with health problems in employment has become increasingly important to compensate for the reduction in labor supply resulting from an ageing population (4).

Vocational rehabilitation (VR) is a commonly used tool to maintain work ability despite health problems. VR can include a range of work-related activities that improve the chances of staying in one’s current job, changing tasks or finding an occupation that is more suitable to one’s health situation. However, the effectiveness of VR is still being debated. While many studies have shown positive effects, in others the effects have been weak or non-existent (5–9). Comparison of the various studies is naturally difficult due to the variation in the content of VR. Another reason for the unclear effectiveness is the strong selection of participants in the VR programs. VR is intended for people who, on the one hand, are at risk of being excluded from working life due to disability, but, on the other, are most likely to benefit from participation (10–12). Self-selection of the most motivated participants in VR is a major issue when evaluating its effectiveness (13, 14). It seems that studies which have been able to better control for such selection have often shown weaker effectiveness than purely observational studies (9, 15).

While randomized controlled trials are hard to conduct in this area of research, some studies have tried to overcome the problem of selection by using quasi-experimental study designs. Propensity score matching has been a commonly used technique (16–18). A recent German study comparing vocational training program participants and drop-outs found that the participants had more working days and higher earnings, less days on social security benefits and lower probability of receiving earnings incapacity pension (19). Another German study, focusing on the unemployed, found better employment prospects among persons accepted to VR than among rejected applicants (20). A Finnish study compared the employment rate among participants and non-participants to VR, matched one-by-one with propensity scores using a large number of demographic factors, work-related characteristics, and detailed labor market histories (21). The study consisted of recently employed individuals aged 30–55 who had suffered from mental disorders or musculoskeletal diseases. VR was found to have a small positive effect (around 10 percentage points) on work participation. By means of matching approaches, these studies have tried to construct a control group that is as similar as possible to the group of people participating in VR. Nevertheless, the participants and non-participants are similar in terms of observed characteristics only, and it remains possible that they differ in some other respects that have not been measured.

In this study, we use another approach to circumvent the problem of selection. Since 2015, disability pension applicants in Finland who are not granted a permanent or long-term disability pension have also received a preliminary decision on VR when their disability pension application is decided. A key requirement for eligibility is that the person must have received a certain amount of income in the previous five years. It can be assumed that disability pension applicants just below and just above this income limit will be similar in their characteristics. We exploit this income requirement to examine the effectiveness of assignment to VR using regression discontinuity design. If we find differences in employment outcomes between the groups at the income threshold, rehabilitation is the possible mechanism that can explain these differences.

## Methods

### Study context

In Finland, rehabilitation is organized by several parties and consists of various rehabilitation sub-systems. Pension insurers, who provide disability pensions, are the primary organizers of VR for people who are attached to employment (22). Most commonly such VR consists of work try-outs in the employee’s previous job or training for new tasks, but it can also consist of learning a new occupation or support for setting up a business. Eligibility to VR requires that one has an illness or injury that is likely to lead to a disability pension within the next five years or so, if no action is taken. Furthermore, it is also based on the expectation that VR can prevent or postpone disability retirement. The pension insurers do not provide medical rehabilitation, and if the person has no recent work history or the disability is due to an occupational or a traffic accident, VR is provided by other organizers.

The VR participants can be currently employed or persons seeking to return to work after an illness. The initiative to apply for VR can come from the employee, the occupational healthcare service, or the employer. If the pension insurer accepts the application, the applicant will receive a preliminary decision for VR, which is valid for 10 months. The rehabilitee and the employer have this time to make the practical arrangements and start the rehabilitation (however, usually the rehabilitation starts shortly after the preliminary decision has been provided). VR based on the rehabilitee’s own application is still the main route to VR, but since the beginning of the 2015, applicants of disability pension have automatically received a preliminary decision for VR if they are eligible for it. A key requirement for eligibility is that the person must have received a certain amount of income during the previous five years (≥€34 910.29 in 2017). If the applicant is granted a permanent full-time disability pension, a preliminary decision for VR is obviously not offered. Similarly, if the applicant is granted a temporary disability pension for ≥10 months, a preliminary decision for VR is not provided as it will expire during the disability pension period. Thus, the preliminary decision for VR concerns those applicants for disability pension who are rejected or who are granted a relatively short (<10 months) disability pension.

### Data sources

**Figure 1:**
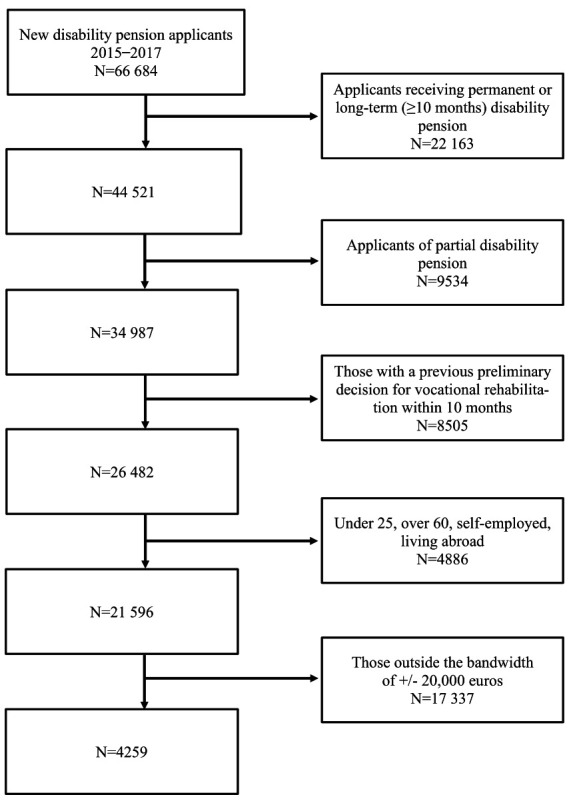
Flowchart of the data formation.

This study is based on the register data from the Finnish Centre for Pensions. The data included all new disability pension applicants from 2015–2017, a new applicant being a person who has not applied for a disability pension or received a pension in the last four years. Applicants receiving permanent or long-term (≥10 months) disability pensions were excluded. Applicants of partial disability pensions were also excluded. In addition, we excluded all those who had already received a preliminary decision for VR within the last 10 months as they would not have received a new decision on their eligibility to VR while the previous one was still valid. A flowchart of the data formation is presented in figure 1.

After further excluding people <25 [due to the difficulty of defining some covariates (N=1644)], people aged >60 [because they have many other ways of leaving employment (N=2591)], self-employed people [for whom VR is inherently different than for wage-earners (N=386)], and people living abroad at the end of the year before the application (N=265), the data included 9718 persons who had received a rejection and 11 878 persons with a positive decision on their disability pension application, totaling 21 596 persons.

The data did not directly include information on whether the preliminary decision for VR had been provided in connection with the decision on disability pension. Therefore, if the disability pension decision and the preliminary decision for VR had been issued within two weeks (+/-14 days) they were considered to have been given together (in most cases the date was the same). Using this definition, 25% of the study population had received a preliminary decision for VR with the decision for disability pension.

### Employment outcomes

The outcome variables in this study were employment, unemployment, and earned income two years after the disability pension decision. Employment and unemployment were measured at the exact date two years after the decision. Unemployment was measured by receipt of basic or earnings-related unemployment benefits. Being defined as employed required an employment contract and not being in receipt of unemployment benefits. Earned income was based on annual income for the calendar year in which two years had elapsed since the decision.

### Statistical methods

To estimate the impact of VR on employment outcomes, we used a quasi-experimental regression discontinuity (RD) design (23–25). The method takes advantage of a clinical or policy decision rule that assigns participants to an intervention or control group based on whether they exceed or fall below a specific threshold value of a continuous assignment variable. It can be assumed that those just above or just below the threshold are similar with respect to unobserved confounders, allowing a causal interpretation of the intervention.

In this study, income was used as the assignment variable and the sum qualifying one to the preliminary decision for VR was used as the threshold value. We did not have access to the amount of income calculated at the time of the disability pension decision, so we had to calculate it ourselves, strictly following the official method of calculation. For those who received a positive decision to their disability pension application, income was calculated from five calendar years before the illness that led to the application, while for those who were rejected, income from the five years preceding the application was used. A wage coefficient was used to convert income from the previous years to the level of the decision year. The income limit is revised annually and was €34 910.29 in 2017. We converted the income from all three study years to the 2017 level, pooled the study years in a single analysis, and centered the income variable at the cut-off value.

**Figure 2:**
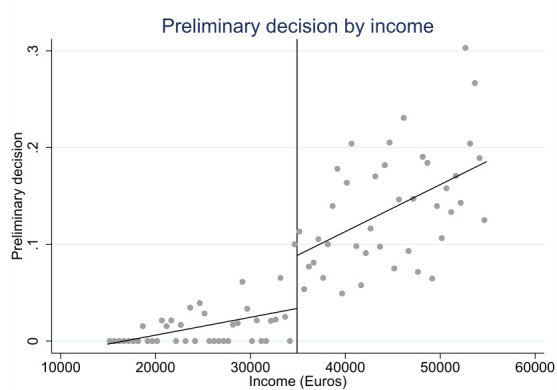
The probability of receiving a preliminary decision for VR by income within the selected bandwidth of +/- €20 000.

The RD design can be divided into “sharp” and “fuzzy” versions (23, 26). The design is sharp if the probability of assignment to the intervention changes deterministically from 0 to 1 at the threshold. In principle, the income limit for the preliminary decision is very strict and it is rigorously adhered to by the decision makers. However, there may be factors related for example to the type of illness that prevent the preliminary decision from being given even if the limit is exceeded. In addition, in some cases it was difficult to ascertain the starting date of the illness that led to the disability pension, which in turn determines the calendar years that are used in calculating the income. Also, there may be changes in the register data over time and therefore information used to calculate the income retrospectively may not give exactly the same result as at the time of the decision. A small proportion of people in the data (<1%) had received a preliminary decision for VR even if their income did not exceed the income limit, indicating that there was some misclassification of income in the data. Therefore, we used the fuzzy RD design which does not require that the assignment to the intervention changes deterministically when the income limit is exceeded but only that the probability of assignment changes at the threshold. Figure 2 shows that this is the case.

The RD design is based on the idea that observations below and above the threshold are similar in observed and unobserved characteristics and do not change abruptly at the threshold. However, if the analyses are restricted very close to the threshold, this limits the sample size and reduces statistical power (24). The choice of an appropriate bandwidth is therefore a trade-off between bias and precision. Furthermore, there are no strict guidelines for deciding what is the optimal bandwidth. For the main analysis, we used a bandwidth of +/- €20 000 for all outcomes. We wanted to omit those with no or minimal income as they may be different from those with incomes even slightly above the threshold. Using this bandwidth, the analyses included 4259 persons, of whom 2364 were in the group below the threshold and 1895 in the group above the threshold. Table 1 shows the comparison of observed demographic covariates in these groups. When the group averages are compared, being employed at time of the disability pensions decision was more common and the length of previous working career was somewhat longer in the group above the threshold. Differences in other characteristics are fairly small, albeit some of them are statistically significant. However, when we compare the groups at the threshold, the differences between the groups disappear. Group averages were tested using chi-square and t-test. The values at the threshold were estimated using the Stata’s margins command and the differences at the threshold were tested by regression models including a dummy variable for exceeding the income limit in addition to continuous income. Plotting the covariates by income deciles on both sides of the threshold also suggest that there is no marked discontinuity at the threshold (supplementary material, www.sjweh.fi/article/4038, figure S1).

**Table 1:** Comparison of the covariates among those below (-€20 000–0, N=2364) and above (€0–20 000, N=1895) the threshold providing eligibility to a preliminary decision for vocational rehabilitation. Average value for the whole group and projected value at the border of the groups estimated within the range of +/- €20 000.

	Group average				P-value of difference	Estimated value at the threshold				P-value of difference
	
	Below the threshold		Above the threshold			Below the threshold		Above the threshold		
			
	%	Mean	%	Mean		%	Mean	%	Mean	
Men	42.8		45.4		0.08	41.8		45.9		0.05
Age		43.2		44.3	0.01		44.2		43.8	0.89
Married	31.2		34.7		0.01	35.1		35.9		0.97
Education					0.18					0.17
Tertiary	26.4		25.3			24.6		28.7		
Secondary	60.5		59.6			61.9		57.1		
Primary	13.2		15.1			13.5		14.1		
Residence					0.01					0.44
Urban	69.5		65.4			67.0		63.5		
Densely populated	15.4		16.5			16.7		17.5		
Rural	15.0		18.5			16.2		18.9		
Employed at baseline	8.6		12.5		<0.001	9.8		9.8		0.97
Working career		10.5		13.7	<0.001		12.2		13.0	0.19

The fuzzy RD design applied in this study is conceptually similar to the instrumental variable (IV) framework (23, 27). With instrumental variables, two-stage least squares (2SLS) models are typically used. In our case, the first stage of the 2SLS model predicts the probability of receiving the preliminary decision for VR while the second stage uses the expected probabilities from the first stage to estimate its impact on employment outcomes. The 2SLS models estimate the complier average causal effect (CACE), which is the effect of the intervention on those who always comply with their assignment into the control or intervention group (26, 27). Since employment and unemployment were measured by dichotomous variables, we used the IV-probit model for these outcomes. For earned income, the corresponding linear model was used. The analyses proceeded in three steps: The first model for each outcome included only the assignment variable (continuous income, allowing for different slopes below and above the threshold) and an indicator variable denoting whether a person exceeded the income limit giving eligibility for VR. Secondly, decision year (2016 or 2017 versus 2015) and a variable describing whether the application for disability pension was granted or rejected were included as design-related control variables. Thirdly, a group of measured covariates (age, gender, marital status, level of education, degree of urbanization of the area of residence, employment status at the time of the disability pension decision, length of working career) were included as additional control variables. Our primary interest is in the indicator variable describing whether the income limit that allows assignment to VR is exceeded, and we do not expect the control variables to markedly affect its value. Confidence intervals (CI) were calculated from the robust 2SLS standard errors. All analyses were performed using Stata version 16.1 (Stata Corp, College Station, TX, USA).

## Results

**Figure 3:**
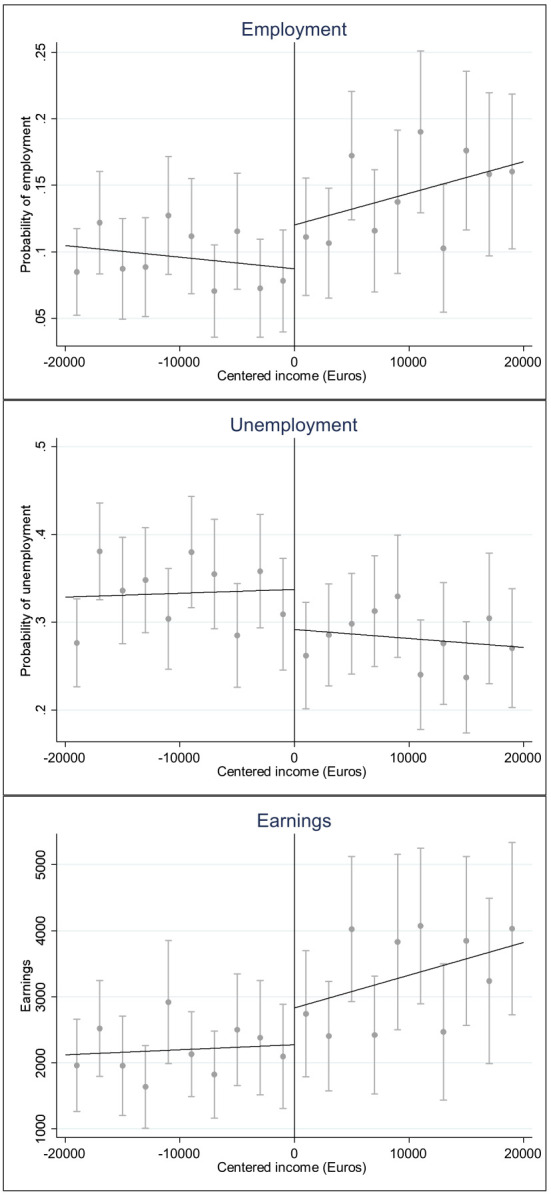
Employment, unemployment and earnings two years after the disability pension decision by income near to the threshold (+/- €20 000) providing eligibility to a preliminary decision for vocational rehabilitation.

Figure 3 shows regression discontinuity plots of the three employment outcomes by income using the bandwidth of +/- €20 000. Each of the employment outcomes show a slight jump in the expected direction at the threshold, suggesting that receiving the preliminary decision for VR is associated with a higher probability of employment and higher income, and a lower probability of unemployment. However, the variation around the regression line is fairly large.

Next, we conducted regression analyses to evaluate the significance of the change in the employment outcomes at the income threshold. Table 2 presents the marginal coefficients of the impact of VR on the probability employment. On average, exceeding the income limit for the preliminary decision of VR increased in the probability of employment by 7.6% percentage points, but the estimate was far from statistical significance. Controls for study design and demographics did not change these results. Incidentally, among the covariates, receiving a rejection to the disability pension application and being of younger age were associated with a higher probability of employment.

**Table 2:** Regression discontinuity estimates for the impact of assignment to vocational rehabilitation on employment after two years. Marginal effects for the probability of employment from IV-probit model. [CI=confidence interval.]

	Model 1	P-value	Model 2	P-value	Model 3	P-value
		
	Estimate (95% CI)		Estimate (95% CI)		Estimate (95% CI)	
Assignment to vocation rehabilitation	0.076 (-0.474–0.627)	0.79	0.077 (-0.458–0.612)	0.78	0.66 (-0.479–0.624)	0.82
Design-based variables						
Decision year (versus 2015)						
2016			0.014 (-0.012–0.040)	0.28	0.011 (-0.013–0.036)	0.39
2017			0.011 (-0.020–0.042)	0.48	0.008 (-0.016–0.039)	0.63
Rejection (versus positive decision)			0.030 (0.011–0.050)	0.002	0.032 (0.012–0.051)	0.005
Covariates						
Women (versus men)					0.028 (0.007–0.051)	0.03
Age (1 year increment)					-0.005 (-0.006–-0.003)	<0.001
Married (versus non-married)					0.024 (0.002–0.046)	0.03
Educational level						
Secondary (versus primary)					0.013 (-0.004–0.034)	0.03
Tertiary (versus primary)					0.059 (0.024–0.094)	<0.001
Residence						
Densely populated (versus urban)					0.007 (-0.015–0.034)	0.63
Rural (versus urban)					0.026 (-0.002–0.055)	0.08
Employed at baseline (versus not employed)					0.214 (0.138–0.290)	<0.001
Working career (1 year increment)					0.002 (0.000–0.004)	0.11

Receiving a preliminary decision of VR resulted in a 3.8% percentage point decrease in the probability of unemployment at the income threshold, but again the estimate was very imprecise (table 3). The inclusion of covariates had some effect on the estimates, suggesting that local randomization was not perfect, but it did not change the main conclusions. Having a rejection and higher age were associated with a higher probability of unemployment.

**Table 3:** Regression discontinuity estimates for the impact of assignment to vocational rehabilitation on unemployment after two years. Marginal effects for the probability of employment from IV-probit model. [CI=confidence interval.]

	Model 1	P-value	Model 2	P-value	Model 3	P-value
		
	Estimate (95% CI)		Estimate (95% CI)		Estimate (95% CI)	
Assignment to vocation rehabilitation	-0.038 (-0.984–0.908)	0.94	-0.033 (-0.939–0.872)	0.94	-0.025 (-0.960–0.927)	0.97
Design-based variables						
Decision year (versus 2015)						
2016			-0.024 (-0.060–0.011)	0.18	-0.023 (-0.058–0.013)	0.22
2017			-0.035 (-0.079–0.009)	0.12	-0.032 (-0.078–0.015)	0.18
Rejection (versus positive decision)			0.302 (0.254–0.350)	<0.001	0.297 (0.249–0.345)	<0.001
Covariates						
Women (versus men)					-0.015 (-0.045–0.016)	0.34
Age (1 year increment)					0.008 (0.005–0.011)	<0.001
Married (versus non-married)					0.004 (-0.026–0.034)	0.79
Educational level						
Secondary (versus primary)					-0.036 (-0.071–-0.002)	0.04
Tertiary (versus primary)					-0.044 (-0.089–0.001)	0.06
Residence						
Densely populated (versus urban)					0.018 (-0.021–0.057)	0.36
Rural (versus urban)					0.010 (-0.028–0.047)	0.61
Employed at baseline (versus not employed)					-0.059 (-0.120–0.001)	0.05
Working career (1 year increment)					-0.007 (-0.012–-0.002)	0.01

Table 4 shows the results for earned income. Receiving a preliminary decision increased the annual income by around €10 000, but the result was again statistically non-significant. A later decision year, rejection of the disability pension application and having a tertiary education were associated with a slightly higher earned income.

**Table 4:** Regression discontinuity estimates for the impact of assignment to vocational rehabilitation vocational rehabilitation on income earned after two years. Estimates of earnings from the linear IV model. [CI=confidence interval.]

	Model 1	P-value	Model 2	P-value	Model 3	P-value
		
	Estimate (95% CI)		Estimate (95% CI)		Estimate (95% CI)	
Assignment to vocation rehabilitation	10171 (-4794–25 137)	0.18	10158 (-4364–24 681)	0.17	9529 (-4851–23 909)	0.22
Design-based variables						
Decision year (versus 2015)						
2016			673 (129–1217)	0.02	566 (41–1091)	0.03
2017			1094 (351–1836)	0.004	952 (208–1697)	0.01
Rejection (versus positive decision)			1100 (677–1522)	<0.001	1233 (801–1665)	<0.001
Covariates						
Women (versus men)					634 (134–1134)	0.01
Age (1 y increment)					-72 (-110–-34)	<0.001
Married (versus non-married)					434 (-23–891)	0.06
Educational level						
Secondary (versus primary)					203 (-264–669)	0.39
Tertiary (versus primary)					1478 (707–2250)	<0.001
Residence						
Densely populated (versus urban)					33 (-507–573)	0.91
Rural (versus urban)					490 (-91–1071)	0.10
Employed at baseline (versus not employed)					3597 (2457–4737)	<0.001
Working career (1 year increment)					-15 (-78–48)	0.65

Sensitivity analyses were conducted using the bandwidth of +/- €10 000 (supplementary table S1). The results remained essentially the same.

## Discussion

VR can play an important role in extending working careers, as people are increasingly expected to work into older ages. Exploiting discontinuity in the probability of receiving a preliminary decision for VR, we used a fuzzy regression discontinuity design to evaluate the impact of assignment to VR on employment outcomes based on nationally representative register data. Our study makes a novel contribution to the literature by using a causally strong study design that, to our knowledge, has not been used previously in this context. For each of the employment outcomes, we found a modest effect in the expected direction at the regression discontinuity threshold, but these results were clearly statistically non-significant.

Detecting a statistically significant association between assignment to VR and the employment outcomes is made difficult by the large variation seen in these outcomes. Being employed two years after the decision was rare on both sides of the income threshold. Participation in VR is presumably the only potential mechanism that could provide differences in employment outcomes between those receiving and not receiving the preliminary decision. However, even if the disability pension applicant receives the preliminary decision, he or she may still decide not to participate. If the participation rate is low, the impact of the preliminary decision remains limited.

Particularly, the lack of a significant effect may be due to the fact that the studied population is limited and specific. It is plausible that the effectiveness of VR is poorer among those who are offered VR when they apply for disability pension than among those who have applied for rehabilitation themselves as their motivation to continue working is likely to be weaker, and they are already oriented to exit the labor market. Furthermore, even among the disability pension applicants the studied population consists of low-income earners. It can be assumed that those with higher incomes have closer connections to the labor market and better chances of employment.

However, the results broadly agree with previous Finnish studies, which have also found relatively modest impact of VR on labor market outcomes. A study using propensity score matching found that shorter rehabilitation was associated with slightly higher work participation during the first year after rehabilitation, while among those with longer rehabilitation, the difference between rehabilitees and non-rehabilitees only emerged during longer follow-up (21). Another report using the same data found that VR reduced the risk of full disability retirement but increased the risk of partial disability retirement, with no difference in expected years of disability pension between the groups (28). An earlier study (29) comparing participants to VR to those with a rejected application or those who interrupted their rehabilitation program found that participation in VR slightly increased the probability of employment in the short term. Over a longer follow-up period, the differences between the employment rates of the rehabilitees and the control group disappeared.

### Methodological considerations

The regression discontinuity design relies on the assumption that, close to the cut-off point, the treatment and control groups do not differ in terms of observed or unobserved characteristics. In our study, employment at baseline was slightly more common and previous working career was somewhat longer among those above the income limit, but the increase by income was fairly monotonic and there was no discontinuity at the threshold.

Due to the limited statistical power, we were not able perform subgroup analyses based on the covariates as is often done in studies based on this method. To evaluate whether our estimates were sensitive to the covariates, we included them in the models, finding that they did not markedly affect the estimate of our key variable: the assignment to VR.

Our study population included participants whose disability pension application was rejected or who received a short-term positive decision. Those who received a rejection were more often employed and had a slightly higher income after two years, but they were also more often unemployed than those with a positive decision. A higher probability of employment among those with a rejection may be explained by their better health as not having serious enough disabilities is one of the most common reasons for rejection. Furthermore, the relatively short follow-up time may have influenced the results. Temporary disability pensions are often continued after the initial period, and some of them are still ongoing after two years (30). Although also those with a rejection to their application may later receive a positive decision, this may be more likely among those with a positive decision. Furthermore, if the participant started VR after a positive preliminary decision, rehabilitation could continue after two years as rehabilitation starts only after the temporary disability pension, when the participant’s state of health has improved. It would therefore be useful to be able to monitor the employment outcomes over a longer period than was possible here.

### Concluding remarks

Our study examined whether receiving a preliminary decision for VR was associated with employment outcomes after two years. If there were differences in employment outcomes between groups below and above the income threshold, VR would be the only conceivable mechanism that could explain these differences. However, we did not find consistent evidence of such an effect. If an effect had been observed, it would have argued for the effectiveness of VR. However, given that our study population is quite limited and specific and the evaluated treatment being the preliminary decision of rehabilitation, we do not believe that the absence of an effect should be taken as evidence of ineffectiveness of VR more generally.

### Conflict of interest

The authors report no conflicts of interest

### Ethical statement

In Finland, register-based studies do not require an ethical review. However, data collection, analysis and reporting were carried out in accordance with the ethical standards of The Finnish Advisory Board on Research Integrity

## Supplementary material

Supplementary material
