# Supplementary material for: The impact of vocational rehabilitation on employment outcomes: A regression discontinuity approach
Source: Scand J Work Environ Health. 2022 Aug 31;48(6):498–506. doi: 10.5271/sjweh.4038 (PMC9888440; doi:10.5271/sjweh.4038)
Supplement: Supplementary material [file SJWEH-48-498-S001.pdf]

The impact of vocational rehabilitation on employment outcomes: A regression discontinuity approach[1]

by Mikko Laaksonen, PhD,[2] *Ilari Ilmakunnas, PhD, Samuli Tuominen, MSc*

1. Supplementary tables and figures
2. Correspondence to: Mikko Laaksonen, Finnish Centre for Pensions, FI-00065 Eläketurvakeskus. [E-mail: mikko.laaksonen@etk.fi]

Supplementary figure S1. Plots of the observed demographic covariates by income in deciles (€2000 each) below and above the income limit giving eligibility to VR

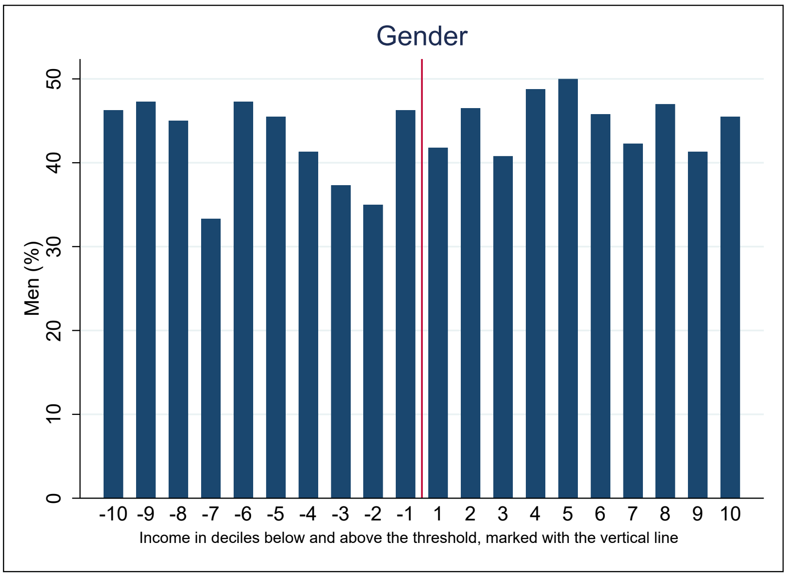

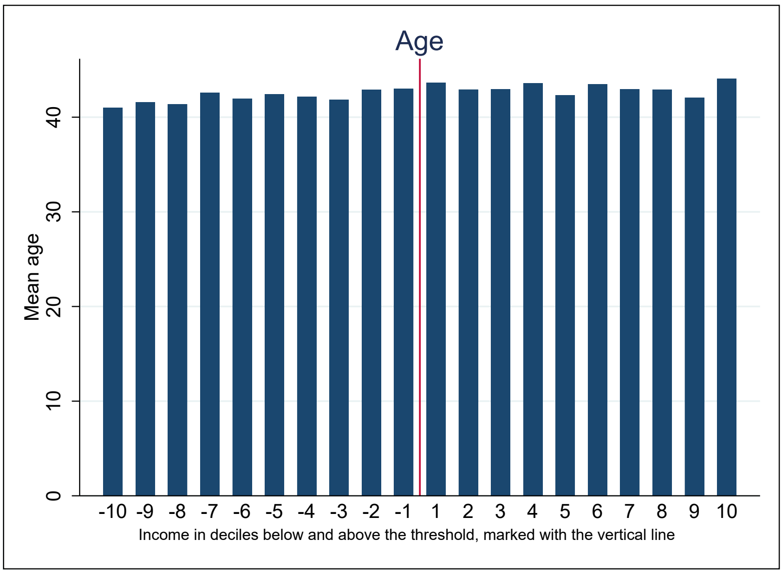

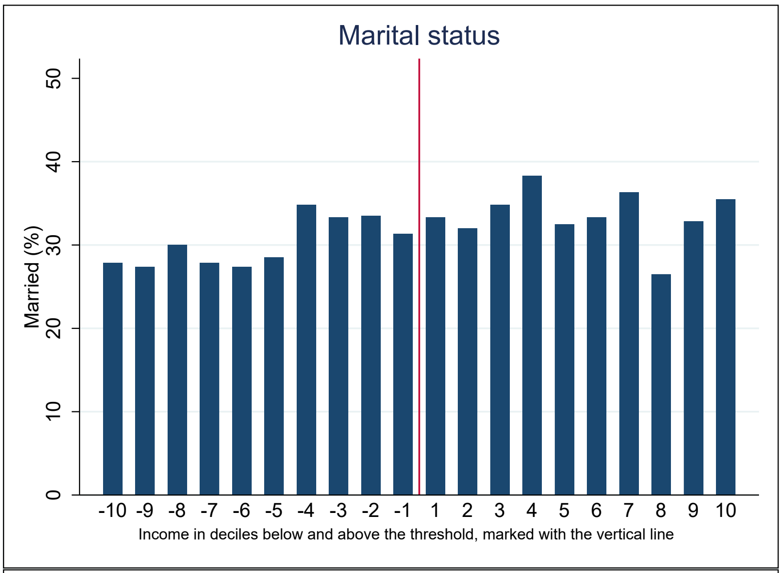
Marital status
Married (%)
0
10
20
30
40
50
-10 -9 -8 -7 -6 -5 -4 -3 -2 -1 1 2 3 4 5 6 7 8 9 10
Income in deciles below and above the threshold, marked with the vertical line

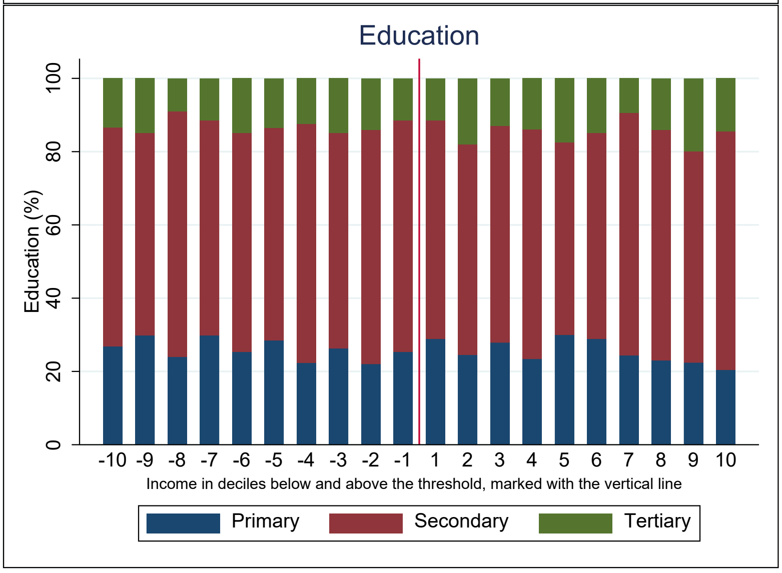
Education
Education (%)
0
20
40
60
80
100
-10 -9 -8 -7 -6 -5 -4 -3 -2 -1 1 2 3 4 5 6 7 8 9 10
Income in deciles below and above the threshold, marked with the vertical line
Primary
Secondary
Tertiary

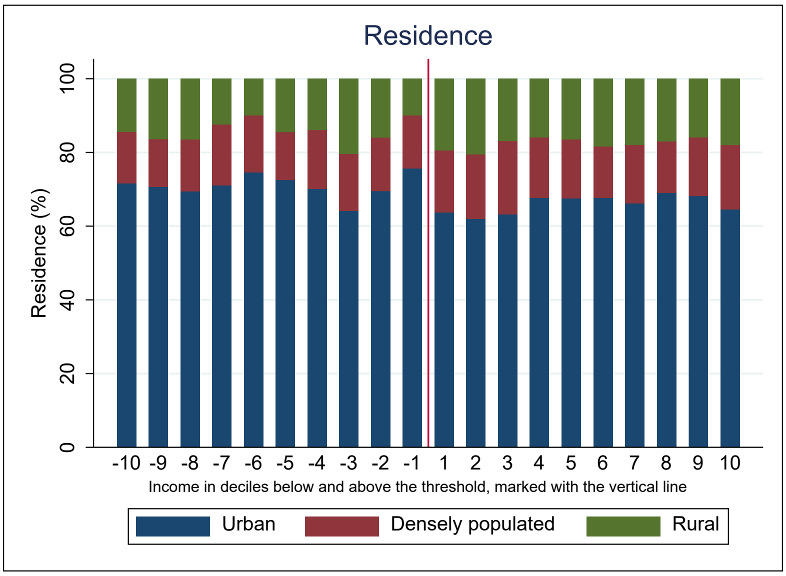
Residence
Residence (%)
0
20
40
60
80
100
-10 -9 -8 -7 -6 -5 -4 -3 -2 -1 1 2 3 4 5 6 7 8 9 10
Income in deciles below and above the threshold, marked with the vertical line
Urban
Densely populated
Rural

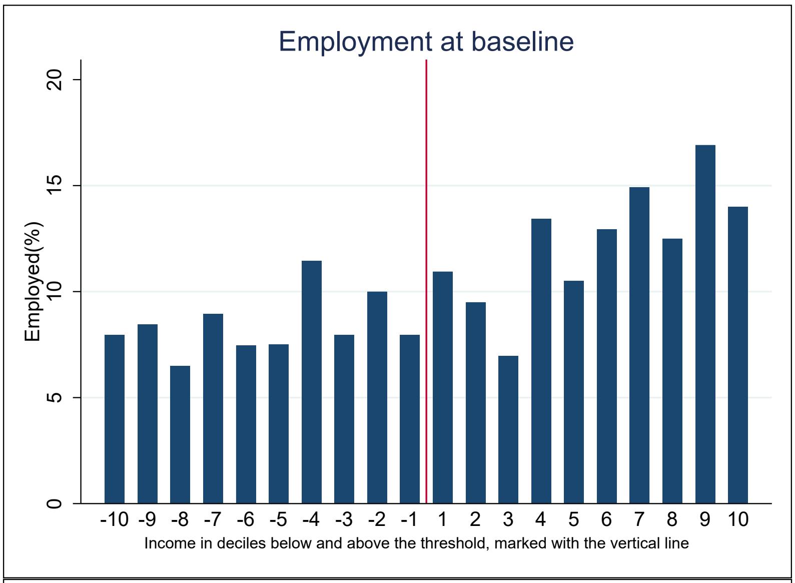
Employment at baseline
Employed(%)
0
5
10
15
20
-10 -9 -8 -7 -6 -5 -4 -3 -2 -1 1 2 3 4 5 6 7 8 9 10
Income in deciles below and above the threshold, marked with the vertical line

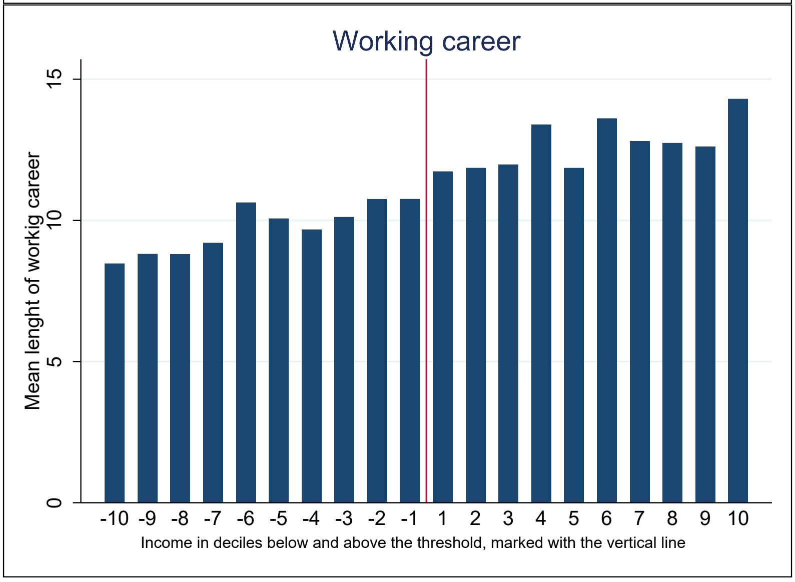
Working career
Mean lenght of workig career
0
5
10
15
-10 -9 -8 -7 -6 -5 -4 -3 -2 -1 1 2 3 4 5 6 7 8 9 10
Income in deciles below and above the threshold, marked with the vertical line

Supplementary table S1. Sensitivity analyses using an alternative bandwidth of +/- €10 000. Group size below the threshold is 1012 and above the threshold 1002

S1a. Regression discontinuity estimates for the impact of assignment to vocational rehabilitation (VR) on employment after 2 years using a bandwidth of €10 000

	Model 1			Model 2			Model 3	
	Estimate (95% CI)	p-value		Estimate (95% CI)	p-value		Estimate (95% CI)	p-value
Assignment to VR	0.065 (-0.622–0.752)	0.85		0.067 (-0.620–0.753)	0.85		0.060 (-0.673– 0.792)	0.88
**Design-based variables**								
Decision year (vs. 2015)								
2016				-0.011 (-0.045–0.023)	0.53		-0.015 (-0.049–0.021)	0.37
2017				0.001 (-0.044–0.046)	0.98		-0.008 (-0.056–0.039)	0.73
Rejection (vs. Positive decision)				0.035 (0.000–0.070)	0.05		0.036 (-0.014–0.087)	0.16
**Covariates**								
Women (vs. Men)							0.027 (-0.005–0.069)	0.11
Age (1 y increment)							-0.005 (-0.008–-0.002)	<0.001
Married (vs. Non-married)							0.034 (0.001–0.069)	0.05
Educational level								
Secondary (vs. Primary)							0.002 (-0.026–0.033)	0.89
Tertiary (vs. Primary)							0.024 (-0.012–0.074)	0.26
Residence								
Densely populated (vs. Urban)							0.007 (-0.030–0.045)	0.42
Rural (vs.Urban)							0.045 (0.003–0.086)	0.03
Employed at baseline (vs. Not employed)							0.219 (0.096–0.323)	<0.001
Working career (1 y increment)							0.001 (-0.001–0.004)	0.33

Table S1b. Regression discontinuity estimates for the impact of assignment to vocational rehabilitation (VR) on unemployment after 2 years using a bandwidth of 10,000 euros

	Model 1			Model 2			Model 3	
	Estimate (95% CI)	p-value		Estimate (95% CI)	p-value		Estimate (95% CI)	p-value
Assignment to VR	-0.018 (-1.813–1.777)	0.98		0.015 (-1.654–1.675)	0.98		0.023 (-0.721–0.759)	0.98
**Design-based variables**								
Decision year (vs. 2015)								
2016				-0.009 (-0.064–0.045)	0.73		-0.004 (-0.061–0.053)	0.89
2017				-0.022 (-0.098–0.054)	0.57		-0.013 (-0.100–0.074)	0.77
Rejection (vs. Positive decision)				0.308 (0.130–0.486)	<0.001		0.305 (0.116–0.495)	0.002
**Covariates**								
Women (vs. Men)							-0.032 (-0.072–0.008)	0.12
Age (1 y increment)							0.007 (0.000–0.014)	0.04
Married (vs. Non-married)							-0.014 (-0.056–0.027)	0.49
Educational level								
Secondary (vs. Primary)							-0.039 (-0.087–0.010)	0.12
Tertiary (vs. Primary)							-0.027 (-0.093–0.040)	0.43
Residence								
Densely populated (vs. Urban)							0.046 (-0.019–0.111)	0.16
Rural (vs.Urban)							-0.004 (-0.060–0.051)	0.87
Employed at baseline (vs. Not employed)							-0.074 (-0.199–0.051)	0.24
Working career (1 y increment)							-0.005 (-0.015–0.005)	0.29

Table S1c. Regression discontinuity estimates for the impact of assignment to vocational rehabilitation (VR) on earner income after 2 years using a bandwidth of 10,000 euros

	Model 1			Model 2			Model 3	
	Estimate (95% CI)	p-value		Estimate (95% CI)	p-value		Estimate (95% CI)	p-value
Assignment to VR	10524 (-19 997–41 046)	0.50		10101 (-18 474–38 676)	0.49		9422 (-17 196–36 039)	0.63
**Design-based variables**								
Decision year (vs. 2015)								
2016				317 (-515–1149)	0.45		151 (-679–980)	0.72
2017				692 (-714–2099)	0.34		389 (-1108–1887)	0.61
Rejection (vs. Positive decision)				1040 (261–1819)	0.01		1149 (190–2108)	0.02
**Covariates**								
Women (vs. Men)							649 (-59–1356)	0.07
Age (1 y increment)							-57 (-126–11)	0.09
Married (vs. Non-married)							582 (-16–1180)	0.06
Educational level								
Secondary (vs. Primary)							261 (-380–902)	0.42
Tertiary (vs. Primary)							898 (-152–1949)	0.09
Residence								
Densely populated (vs. Urban)							-208 (-896–480)	0.55
Rural (vs. Urban)							809 (-66–1684)	0.07
Employed at baseline (vs. Not employed)							3546 (1684–5407)	<0.001
Working career (1 y increment)							-46 (-172–79)	0.47
